# Book on the Physician Himself

**Published:** 1892-10

**Authors:** 


BOOK ON THE PHYSICIAN HIMSELF, AND THINGS THAT CONCERN HIS REPUTATION AND SUCCESS.—By D. W. Cathell, M. D. New tenth edition (author's last revision). Thoroughly revised, enlarged and rewritten. In one handsome Royal Octavo volume. 348 pages. Bound in extra cloth. Price, post-paid, $2.00, net. Philadelphia: The F. A. Davis Co., Publishers, 1231 Filbert street.

A BOOK, without which the library of every young physician is incomplete; one which the "younger members of our profession and also the older ones who have paused at less than the average degree of success in life," should especially read, but which will be found by all true worshippers at the shrine of Æsculapius to be replete with advice upon the many little points connected with the practice of medicine, a thorough knowledge of which is so necessary to his success.

